# Role of Polymerase Chain Reaction-Based Diagnosis of Respiratory Viruses in Febrile Neutropenic Patients

**DOI:** 10.7759/cureus.33314

**Published:** 2023-01-03

**Authors:** Janani Madhuravasal Krishnan, Dhaarani Jayaraman, Adarsh Kancharla, Aishwarya Thangam, Padmasani Venkatramanan, Julius Xavier Scott

**Affiliations:** 1 Microbiology, Medical Research Foundation, Chennai, IND; 2 Pediatrics, Sri Ramachandra Institute of Higher Education and Research, Chennai, IND

**Keywords:** respiratory infection, sars-cov-2 (severe acute respiratory syndrome coronavirus -2), polymerase chain reaction, respiratory viruses, febrile neutropenia

## Abstract

Background

Neutropenic patients are commonly affected by respiratory infections, whereas respiratory viral infections causing high morbidity and mortality are routinely diagnosed in developing countries like India. Our study aimed to investigate the prevalence of respiratory viral infections in pediatric cancer patients with febrile neutropenia.

Methods

This prospective study was performed on 45 neutropenia patients with hematological malignancies. Nasal swabs were collected and analyzed by real-time multiplex polymerase chain reaction (PCR), covering the following viruses: influenza A virus, influenza B virus, human parainfluenza virus (subtypes 1-4), human respiratory syncytial virus A and B, enterovirus, human-coronavirus (HCoV: HKU1, NL63, 229E, and OC43), human bocavirus, adenovirus, human rhinovirus, human-metapneumovirus A and B, human paraechovirus, and a bacterium *Mycoplasma pneumoniae*. Patients enrolled in the study since the COVID-19 pandemic was also detected for the presence of severe acute respiratory syndrome coronavirus 2 (SARS-CoV-2).

Results

Of the 45 cases included in our study, 26 cases showed the presence of at least one positivity by PCR (57.7%): 23 patients had monoinfection with only one virus, two patients were found positive for coinfection with two viruses, and one patient was found positive for three viruses. The most detected viruses were human rhinovirus (26.9%, n=7) and coronavirus 19 (19.2%, n=5). A total of 11.5% of the patients had multiple viral infections. About 19 (42.2%) of the patients enrolled in our study had no viral pathogen detected.

Conclusion

We found that respiratory viruses contribute significantly to the development of neutropenic fever, as evidenced by the results of our prospective study. Individualizing infection treatment can reduce antibiotic use in immunocompromised patients. Thus, routine screening for viremia may be warranted in this clinical setting.

## Introduction

The condition of febrile neutropenia (FN) occurs frequently in children who undergo chemotherapy [1]. The conditions of fever and neutropenia are frequently associated with anticancer treatment and can lead to potentially fatal infections [2]. Empirical antimicrobial treatment is the key to the successful management of FN [3]. Optimal empiric treatment depends on understanding FN pathogens, local antibiotic susceptibility patterns, and clinical course [1,3]. In view of the difficulty in determining the presence of septicemia (delay may be fatal), a broad-spectrum antibiotic is promptly given at the onset of fever as a result of a wide range of indications [2]. In most cases, the fever does not have any identifiable underlying cause [3]. Long hospitalizations are associated with febrile neutropenia, causing the child's and family's social well-being to be negatively affected [4-6]. Several guidelines for management have been published, but there are very few studies, mainly focusing on bacterial infections but not on viral infections caused by respiratory viruses (RV) [7-9]. Additionally, hospitalization and broad-spectrum antibiotics increase the risk of subsequent antibiotic-resistant infections caused by resistant bacteria and fungi [10-13]. It is, therefore, imperative to treat febrile neutropenia to reduce unnecessary hospitalizations and the associated costs and excessive use of antibiotics. Prompt microbiological identification of the specific causative pathogen could help the clinician in the detection and appropriate treatment to improve the outcome. In recent years, significant progress has been implemented in molecular techniques for the detection of respiratory viral pathogens as compared to conventional methods of cell culture or antigen detection [4,5,14-17]. The present study focused on molecular identification of the respiratory viral pathogen in samples collected from pediatric neutropenic patients presented with fever and respiratory illness.

## Materials and methods

This was a prospective study including a total of 45 FN patients with hematological malignancies who were followed up in a pediatric hemato-oncology unit at a tertiary care center from January 2020 to August 2021. All the patients included in the study were less than 18 years presented with fever and neutropenia (an absolute neutrophil count (ANC) of less than 1500 cells/mm^3^) either with respiratory illness or any of underlying conditions, such as children with solid tumors and hematological malignancies undergoing chemotherapy or radiotherapy or both, immunocompromised with hematological conditions including autoimmune hemolytic anemia, thrombocytopenia on steroids or rituximab/cyclosporine/mycophenolate mofetil and aplastic anemia, primary immunodeficiency, and post-bone marrow transplant.

Following clinical stabilization, a detailed secondary assessment for the clinical focus of infection, including sinus tenderness and perineal focus, was carried out in accordance with the febrile neutropenia protocol. Prior to starting antibiotics, all children's blood samples were given for routine blood cultures (peripheral and central lines as applicable) and hemograms according to unit policy. Urine analysis and culture, as well as imaging studies, were performed when indicated. Real-time multiplex polymerase chain reaction (RT-PCR) studies were conducted on nasal swabs after informed consent was obtained from the patient's parents or guardians.

Initial ethical approval was obtained before starting the study to test the above-mentioned viruses. Later in 2020, the study was amended to include the severe acute respiratory syndrome coronavirus 2 (SARS-CoV-2) virus in light of the SARS-COVID-19 pandemic.

A complete history and physical findings (including symptoms and duration), laboratory parameters (complete blood count (CBC), culture, and viral PCR), treatment details (including the drugs used), reports of investigations, adverse events such as intensive care monitoring and oxygen (O_2_) requirement, as well as the duration of hospitalization, were recorded and analyzed.

Nasal swab specimens were collected from all patients after obtaining informed consent. The samples were collected in a transport medium (HiMedia's HiViral^TM^ Transport Kit with cryoprotectant, HiMedia Laboratories, Nashik, India) and stored at 4 °C and transported within two hours of collection to a referral laboratory for viral RT-PCR in accordance with cold chain protocols. 

Complete nucleic acid extraction from specimens was first performed using the QIAamp MinElute Virus Spin Kit (QIAGEN, Hilden, Germany) according to the manufacturer’s instructions. An input volume of 200 μl of the sample was utilized with an elution volume of 100 μl. The FTD Respiratory Pathogens 21 Kit (Fast-track Diagnostics Ltd., Luxembourg), working with real-time and multiplex PCR, was applied in accordance with the manufacturer’s instructions to detect respiratory tract pathogens on the Rotor-Gene Q platform (QIAGEN, Hilden, Germany). All samples were analyzed for real-time multiplex PCR covering the following viruses: influenza A virus, influenza A (H1N1) virus, influenza B virus, human parainfluenza virus (PIV) (1-4), human respiratory syncytial virus A and B (RSV), enterovirus (EV), human-coronavirus (HCoV: HKU1, NL63, 229E, and OC43), human bocavirus (hBoV), adenovirus (AdV), human rhinovirus (HRV), human-metapneumovirus A and B, human paraechovirus (HPeV), and one bacterium *Mycoplasma pneumoniae*.

This multiplex one-step real-time PCR test combines 5′ nuclease technology with differentially labeled fluorogenic probes. Five microliters of the template were used for each of the five multiplex PCR assays. An internal control (equine arteritis virus, EAV), which was included in the FTD kit, was added to one of the master mix. Thermocycling conditions were as follows: 50 °C for a 15-minute hold and 95 °C for a one-minute hold, followed by 40 cycles of 95 °C for eight seconds and 60 °C for one minute. A kit-provided negative control and positive control were included with each PCR assay. The run is considered valid, and the values of the test samples are considered only when the internal control and positive control show a positive amplification below a cycle threshold (ct) of 33 and the negative control is below the threshold.

Patients enrolled in the study since March 2020 were also tested for severe acute respiratory syndrome coronavirus 2 (SARS-CoV-2) as a routine workup due to the pandemic.

## Results

This is a prospective study that included 45 patients who developed neutropenia and fever during the study period. The study population included 26 boys and 19 girls.

Non-Hodgkin's lymphoma was the most common diagnosis majorly B-cell acute lymphoblastic leukemia (ALL). The detailed patient characteristics are shown in Table 1. Most patients were treated with chemotherapy (84%) followed by 8.8% bone marrow transplant recipients and 6.6% requiring immunosuppressive therapies with primary immunodeficiencies.

**Table 1:** Details of the underlying diagnosis and respiratory virus detection.

Underlying condition	Patients (n)	One virus detected	Multiple viruses detected
Acute lymphoblastic leukemia	3	2	0
Acute myeloid leukemia	1	0	0
Hodgkin’s lymphoma	2	1	1
Non-Hodgkin’s lymphoma	18	8	1
Solid tumors	14	8	1
Post-bone marrow transplant	3	2	0
Aplastic anemia	4	2	0
Total	45	23	3

The most common symptom found in our study group was fever, followed by respiratory tract complaints, which included rhinorrhea, coughing, sneezing, and sore throat. Among 37.7% of patients who presented with respiratory complaints, 52.9% had lower respiratory tract illness, and 47.05% were involved in the upper respiratory tract. The clinical characteristics and outcomes are listed in Table 2.

**Table 2:** Clinical characteristics and outcomes. PICU: pediatric intensive care unit.

Clinical characteristics	Results
Median age	7.7 Years
Boys	26/45 (57.7%)
Girls	19/45 (42.3%)
Fever	41 (91.2%)
Respiratory symptoms (cough/coryza/sore throat)	17 (37.8%)
Fever + respiratory symptoms	14 (31.1%)
Other complaints, diarrhea, vomiting, myalgia, arthralgia	9 (20%)
Respiratory distress and PICU stay	7 (15.5%)
Profound neutropenia (absolute neutrophil count <100 cells/cubic mm)	18 (40%)
Duration of illness >7 days	11/45 (24.4%)
Mortality	1/45 (6.7%)

Of the 45 cases included in our study, 26 cases showed the presence of at least one positivity by PCR (57.7%): 23 patients had monoinfection with only one virus (seven with rhinovirus, five with coronavirus-19, two with coronavirus 229E and two patients with influenza A H1N1 followed by influenza A, influenza B, parainfluenza 1, parainfluenza 2, paraechovirus, and adenovirus in one patient only). Two patients were found positive for coinfection with two viruses. Rhinovirus and adenovirus in one patient and rhinovirus with parainfluenza-3 in another patient. One of the patients was found positive for three viruses with coronavirus 229, RSV, and enterovirus. The most detected viruses were human rhinovirus (26.9%, n=7) and coronavirus 19 (19.2%, n=5). A total of 11.5% of the patients had multiple viral infections. About 19 (42.2%) of the patients enrolled in our study had no viral pathogen detected.

Blood culture for bacterial isolation was detected positive in six cases, wherein three cases had no virus detected, and three were positive along with viral infection. *Klebsiella pneumoniae* was grown in two patients and *Escherichia coli* in one case with viral PCR negative. In patients with virus positive *Staphylococcus aureus* was grown in blood culture for one patient with viral coinfection, followed by Acinetobacter and *Bacillus cereus* in two other patients with virus positive. No significant difference was noted across the age group in terms of viral PCR positivity.

Details of clinical characteristics and outcomes are depicted in Table 2. In our cohort, 7/45 children (15%) had respiratory distress and required pediatric intensive care monitoring and management, including oxygen support and non-invasive/invasive ventilation. Profound neutropenia (ANC <l00 cells/cubic mm) was noted in 40% of the cohort. One child succumbed to a severe illness in our cohort. Prolonged illness, defined as more than seven days of total illness, was noted in 24.4% of children. The average duration of stay was 6.7 days.

## Discussion

Our study demonstrates the effectiveness of PCR-based viral diagnostics for the detection of infectious agents during febrile neutropenia episodes in children. Higher detection rates were attributed to the addition of molecular-based assays for detecting recently described viruses such as coronavirus 19 and viruses that are difficult to detect by conventional methods. Children with a suppressed immune system need to be diagnosed correctly to tailor the treatment, as it can have serious consequences due to the increased risk of infections caused by viruses [17,18], bacteria [19], and fungi [20]. Though there are several studies on respiratory viruses in children with cancer having febrile neutropenic episodes, there are very limited reported studies on the detection of viruses in febrile neutropenia cases in India by PCR. Hence, we designed this study to collectively analyze the PCR positivity of the virus and correlate it with the respiratory illness of patients with febrile neutropenia.

The majority of studies that investigate febrile neutropenia in children examine bacterial organisms as the cause. Respiratory viruses have, however, been identified as contributing to these illnesses. In a study conducted by Mottonen et al., children with ALL had a higher rate of viral infections than healthy controls but recovered [21,22]. El-Mahallawy et al. found that 13 of 30 cancer patients with lower-respiratory tract symptoms had respiratory virus infection by serological testing [23]. Based on a retrospective chart review over 15 years, Mendoza Sanchez et al. found that 12% of febrile children receiving anticancer therapy were infected with respiratory viruses [24]. Arola et al. identified respiratory viruses in 37% of febrile cancer patients by detecting virus antigens and cultures [25]. Koskenvuo et al. showed that 44% of a similar cohort of patients had a respiratory virus infection [26].

In a study designed to examine the cause of febrile neutropenia in 25 children with acute myeloid leukemia (AML), Täger et al. diagnosed respiratory viruses in 25% of the episodes [27]. The viruses that were detected are influenza, PIV, and RSV, followed by one case of adenovirus, co-infections of PIV + RSV and PIV + adenovirus. Another study by Suryadevara et al. detected respiratory viruses in 26 (52%) febrile episodes. Twenty-two febrile episodes were positive for a single virus, and multiple viruses were detected in the remaining four episodes [6].

In most cases, rhinovirus/enteroviruses and rhinoviruses [26,28] have been isolated. Approximately 5% of isolates of respiratory viruses are coronaviruses [28]. Coronavirus-19 was detected as positive in 19.2% (n=5) of cases in our study. The effect of the human coronavirus on immunocompromised children is not fully understood, despite the fact that it is considered a common cause of upper respiratory tract infections and lower respiratory tract infections in immunocompromised patients.

Routine screening for respiratory disease is not warranted, however, viral testing is helpful in identifying the etiology, establishing barrier precautions, and offering adequate treatment to individuals with respiratory symptoms. A few caveats of our study include a small group of participants and only covering a short period of time. Our findings cannot be directly extrapolated to future viral respiratory seasons in other clinical settings since the circulation and pathogenicity of viruses vary from year to year. Secondly, since the new pandemic SARS-COVID-19 started, there has been a much greater awareness of respiratory virus prevention measures among staff and the general population, such as hand hygiene and vaccination, that may have prevented other respiratory viruses from spreading.

This study documented the role of viral agents detected in 57.7% of pediatric FN cases. The most commonly detected virus in our study was human rhinovirus (26.9%, n=7) and coronavirus 19 (19.2%, n=5).

## Conclusions

The results of our prospective study suggest that respiratory viruses contribute significantly to the development of neutropenic fever, which could lead to individualized infection treatment and the reduction of antibiotic use in immunocompromised patients with neutropenia.
